# Supplementary material for: Photodynamic Therapy-Based Dendritic Cell Vaccination Suited to Treat Peritoneal Mesothelioma
Source: Cancers (Basel). 2020 Feb 27;12(3):545. doi: 10.3390/cancers12030545 (PMC7139796; doi:10.3390/cancers12030545)
Supplement: Supplementary file 1 [file cancers-12-00545-s001.pdf]

Supplementary figures.

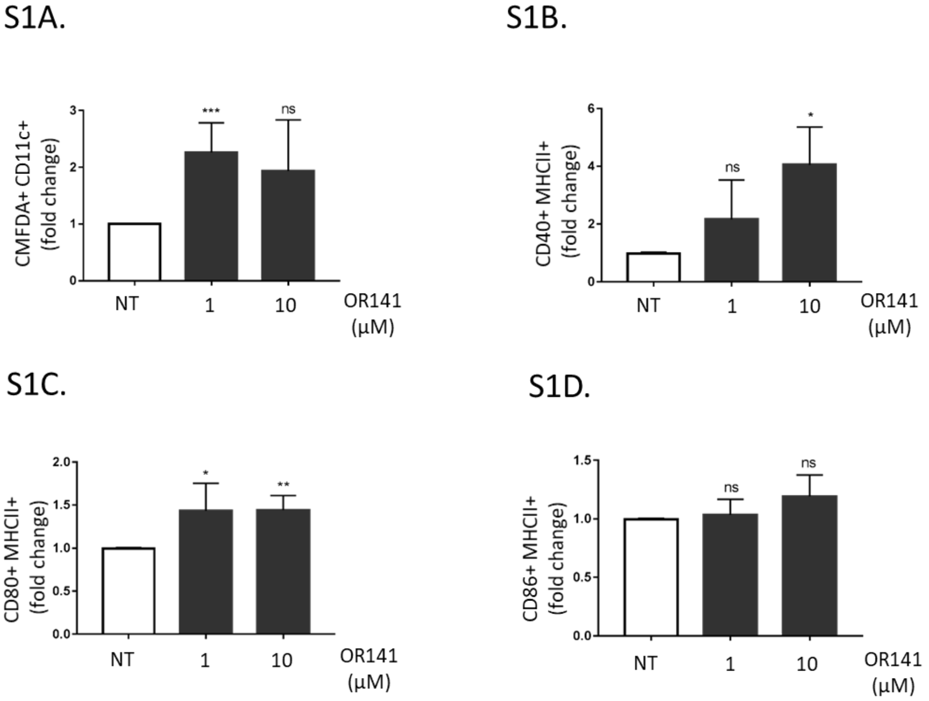

Figure S1. PDT-based DC vaccination leads to potent mesothelioma growth inhibitory effects. Naïve DC were exposed to lysates from OR141-killed Ab12 mesothelioma cells for 18 h. **A.** Phagocytotic activity was evaluated based on the increased incorporation of CMFDA-labelled Ab1 in CD11c$^+$ DC (identified by flow cytometry) (n=3). **B-D.** Extent in CD40$^+$/MHCII$^+$ (**B**), CD80$^+$/MHCII$^+$ (**C**) and CD86$^+$/MHCII$^+$ (**D**) DC populations as determined by flow cytometry (n=3); *P<0.05, **P<0.01, ***P<0.001, n-s, non-significant.

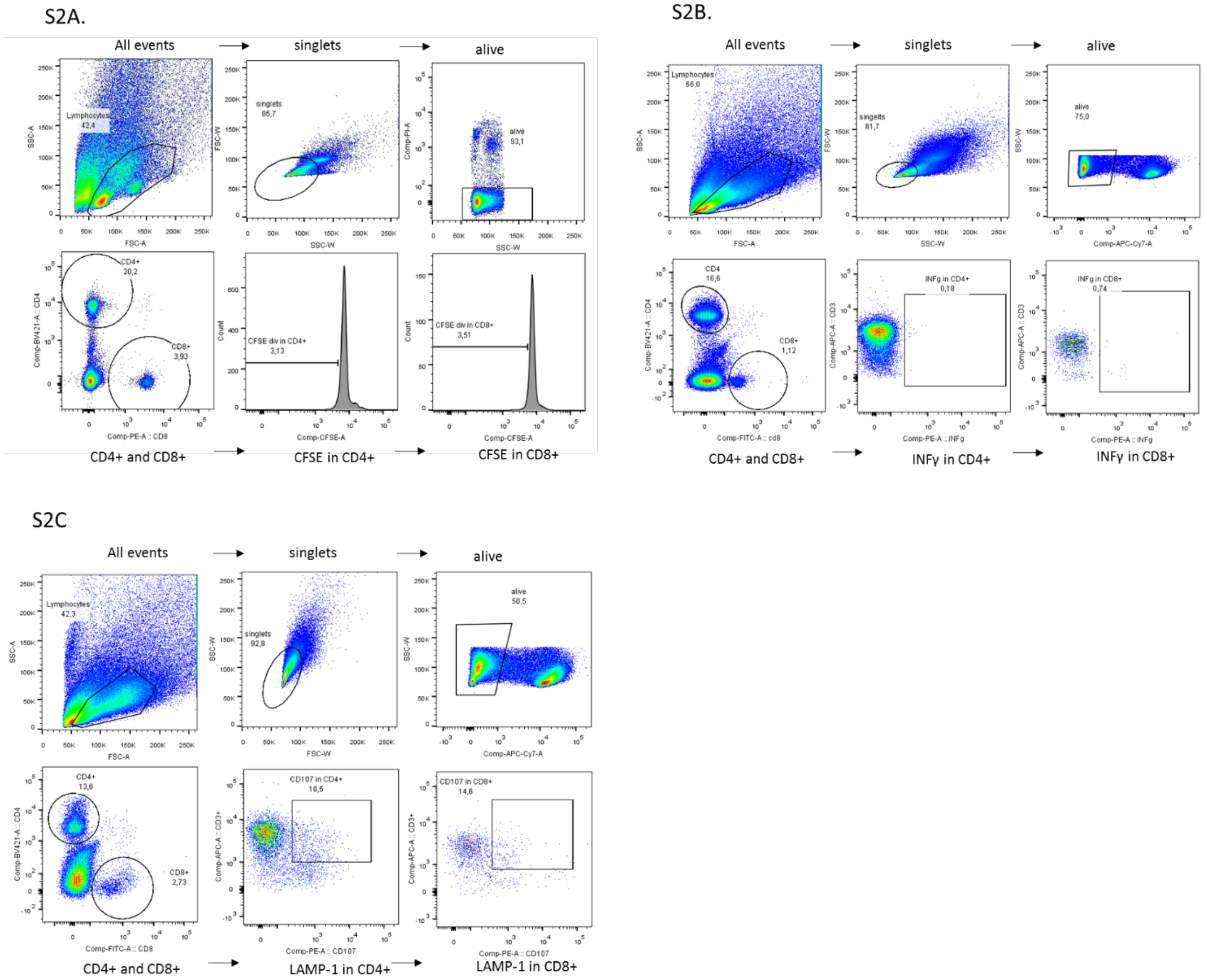

Figure S2. Gating strategies. A. CFSE-based proliferation in CD4$^+$ and CD8$^+$ populations. **B.** INF-γ in CD4$^+$ and CD8$^+$ populations**. C.** LAMP-1 in CD8$^+$ population.

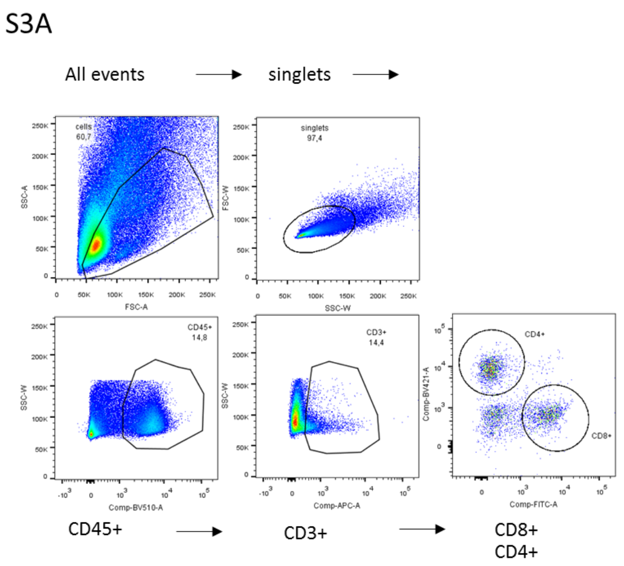

Figure S3. Gating strategies. A. Intratumoral $CD45^{+}$/$CD3^{+}$/$CD8^{+}$ detection.
